# Metabolic Bone Disease in Premature Neonates: An Unmet Challenge

**DOI:** 10.4274/jcrpe.galenos.2019.2019.0091

**Published:** 2020-11-25

**Authors:** Swathi Chacham, Rachna Pasi, Madhuradhar Chegondi, Najeeb Ahmad, Shanti Bhusan Mohanty

**Affiliations:** 1 All India Institute of Institute of Medical Sciences, Rishikesh, India; 2 Himalayan Institute of Medical Sciences, Dehradun, India; 3 University of Iowa, Carver College of Medicine, Iowa City, USA

**Keywords:** Extremely premature, hypocalcemia, hypophosphatemia, neonate, osteopenia, premature, rickets, very low birth weight

## Abstract

Metabolic bone disease (MBD) is an important cause of morbidity in premature, very low birth weight (VLBW) and sick infants and, if left undiagnosed, may lead to structural deformities and spontaneous fractures. MBD is defined as impaired bone mineralization in a neonate with lower than expected bone mineral levels in either a fetus or a neonate of comparable gestational age and/or weight, coupled with biochemical abnormalities with or without accompanying radiological manifestations. MBD has been reported to occur in 16% to 40% of extremely low birth weight neonates and presents by 6-16 weeks after birth. Insufficient calcium and phosphorous stores during the phase of accelerated growth predispose to MBD in neonates along with the use of some medications such as caffeine or steroids, prolonged parenteral nutrition and chronic immobilization. Enhanced physical activity in preterm infants facilitates bone mineralization and weight gain. Biochemical abnormalities tend to worsen significantly, as the severity of disease progresses. These abnormalities may include hypocalcemia, hypophosphatemia, hyperphosphatasia and secondary hyperparathyroidism. In addition, urinary phosphate wasting and hypovitaminosis D can be additional complications. Conversely, biochemical abnormalities may not be accompanied by rachitic changes. Newer diagnostic modalities include non-invasive bone densitometry by quantitative ultrasound over the mid-tibial shaft. The management of MBD includes adequate calcium, phosphorous and vitamin D supplementation, along with optimum nutrition and physical activity. Similarly, preventive strategies for MBD should target nutritional enhancement in combination with enhanced physical activity. MBD usually results in preventable morbidity in preterm and VLBW neonates. Treatment consists of optimum nutritional supplementation and enhanced physical activity.

## Introduction

Metabolic bone disease (MBD) in neonates is associated with reduced bone mineral content (BMC) leading to impaired skeletal mineralization. It is also known as osteopenia of prematurity and is a common consequence of numerous nutritional and biomechanical factors in premature neonates. BMC is inversely proportional to gestational age and birth weight and is influenced by the adequacy of calcium and phosphorus intake in postnatal life (1,2). MBD may or may not be accompanied by rachitic changes (3,4). Although advanced neonatal intensive care has led to improved survival of extremely preterm infants, this has not resulted in the abolition of morbidity and achieval of optimum growth (5,6,7).

MBD is defined as decreased bone mineralization in neonates when compared to *in utero* or *ex utero* bone mineral density of neonates with equivalent gestational age or birth weight along with biochemical evidence and or radiological findings (8,9).

## Magnitude of the Problem

It has been reported that 55% of infants with extremely low birth weight (ELBW) (ELBW ≤1000 grams birth weight) and 23% of infants with very LBW (VLBW) (VLBW <1500 and >1000 grams) have MBD. Similarly, it is more frequent in neonates under 28 weeks of gestation (10,11). The incidence of MBD in breastfed, premature infants is 40% and in formula-fed, preterm infants (with oral calcium and phosphorus supplements) is 16%. As the proportion of extremely preterm and ELBW neonates is increasing, the incidence of MBD is on the rise. Osteopenia occurs in about 50% of VLBW neonates and the majority of ELBW infants at 40 weeks of post conceptional age, without adequate calcium and phosphorous supplementation (10,11,12,13,14,15). It could be more prevalent in both developing and developed countries as more and more sick preterm neonates are being catered with enhanced survival (5,14,15).

## Etiology

MBD is often multifactorial (see Table 1). The leading causes are inadequate mineralization and include intrauterine growth restriction, prolonged parenteral nutrition (PN) (without phosphate) and delayed enteral nutrition. Neonates with insufficient calcium, phosphorous and vitamin D intake are at further risk of MBD when subjected to extended periods of immobilization (3,4,5,6,7,8,9,10,11,12). The onset of MBD ensues between the 6^th^ and 16^th^ week of life or by 40 weeks of corrected age, although it may go unnoticed until marked demineralization takes place (loss of 20-40% of BMC). The physiological basis of MBD is inadequate calcium and phosphorus stores in the face of accelerated fetal growth during the third trimester. *In utero *calcium and phosphorus accretion occurs at the rate of 120 mg/kg/day and 60 mg/kg/day, respectively (15,16). However, impaired supplementation and absorption of these minerals in post natal life leads to a sub-optimally mineralized new and remodelled skeletal system. Preterm, human milk has insufficient calcium, phosphorous and vitamin D *per se*, necessitating supplementation. Vitamin D concentration in human milk is 25 to 50 IU/L which is grossly insufficient to maintain serum 25-hydroxyvitamin D [25(OH)D] levels greater than 20 ng/mL in premature infants. This vitamin D deficiency leads to hypocalcemia, secondary hyperparathyroidism which in turn leads to phosphaturia. In addition, undue fluid restriction, use of term formula, and soy-based and lactose-free formulas in preterm neonates can contribute to MBD (17,18,19,20,21). However, VLBW neonates can produce adequate 1,25-dihydroxyvitamin D levels after the initial few weeks of life if they have optimum dietary vitamin D supplementation. Some medications used in preterm infants including frusemide, steroids and methyl xanthines, which enhance osteoclastic activity, decrease osteoblastic proliferation, reduce calcium absorption and promote renal calcium wasting may lead to osteopenia (19,20,21). Similarly, extended administration of phenobarbital or phenytoin in neonates with seizures can lead to enhanced 25(OH)D metabolism and osteopenia (21).

Maternal vitamin D deficiency often occurs when lactating mothers have inadequate vitamin D (less than 600 IU/day) supplementation. This often manifests as neonatal hypocalcemia and can result in congenital rickets. Neonates with cholestatic liver disease may have exaggerated malabsorption and impaired 25(OH)D, which further aggravates osteopenia. Rare causes of hypovitaminosis D, such as hereditary pseudovitamin D deficiency type 1, due to abnormal or absent 1-α-hydroxylase activity or type 2 due to 1,25-dihydroxyvitamin D resistance in tissues, can also lead to MBD (21). Chronic renal failure can also result in renal osteodystrophy and osteopenia.

Maintaining calcium and phosphorus levels in PN is difficult due to restricted solubility and temperature lability. Aminoacid, glucose, lipid concentration, pH and methods of preparation of calcium salts determine the bioavailability of calcium and phosphorus. Lowering the pH with cysteine enhances solubility.

## Pathophysiology

### Calcium and Phosphorus Homeostasis

The structural matrix of the skeletal system largely made up of calcium, phosphorus and magnesium and their homeostasis play a key role in bone integrity. The majority of total body calcium (99%) and phosphorus (80%) are present in bone as microcrystalline hydroxyapatite. The remainder of total body calcium (1%) lies within the extracellular fluids and soft tissues. However, only 50% of total serum calcium is biologically active as being in the ionized form. The remaining calcium is bound to proteins (albumin and globulin: 40%) and the rest (10%) to organic and inorganic acids. Similarly, a major proportion of magnesium (60%) is present in the bone matrix. Numerous factors such as vitamin D, parathyroid hormone (PTH), and calcitonin, followed by dietary calcium and phosphorous content, intestinal absorption, bone accretion, resorption and, finally, rate of urinary excretion determine calcium and phosphorus homeostasis (15).

### Role of PTH

Soon after the birth, irrespective of gestational age and persisting mineral requirement, there is a fall in calcium, with a nadir attained at 24-30 hours after birth in preterm infants. As a result, there is a PTH surge. PTH augments calcium reabsorption in the kidney and also results in urinary phosphate wasting. PTH aids in the production of calcitriol [1,25(OH)_2_D] by activating renal 25(OH)D_3_-1-alpha-hydroxylase, which increases intestinal calcium and phosphate absorption. PTH also promotes bone resorption and subsequent release of calcium and phosphate. On the whole, PTH has the greatest action in the kidney for regulating calcium metabolism. When there is insufficient calcium intake for prolonged periods, as with MBD, these metabolic changes persist (5).

### Fetal Bone Homeostasis

The amount of minerals required for proper accretion of the skeleton vary according to the age of the baby. The fetus has a higher rate of skeletal growth, especially during the last trimester. There is an enormous increase in bone volume with advanced gestational age due to bone remodelling and augmented bone synthesis as seen by increased trabecular thickness. It has been shown that the rate of trabecular thickening is 240 times more rapid in the fetus compared to children (15). Fundamentally, osteoblasts produce osteoid/organic bone matrix into which calcium and phosphate hydroxyapatite are incorporated. This osteoblastic activity increases exponentially, involving 80% of mineral accretion, during the period of 24 to 37 weeks of gestation (22,23,24,25,26).

Normally the fetal nutrient supply of protein, energy and minerals is ample for fetal growth and skeletal development (1.2 cm/week). The physical density of bone (expressed as bone mass divided by bone volume) is highest in term neonates. The calcium and phosphate deposition during the last trimester of fetal life is around 20 grams and 10 grams respectively, which corresponds to a calcium and phosphate accretion rate of 100-120 mg/kg/day and 50-65 mg/kg/day respectively (15,16).

The placenta has a pivotal role in fetal skeletal development as calcium is actively transported transplacentally with the aid of a calcium pump in the basement membrane (22,23,24,25,26) with a maternal to fetal calcium gradient of 1:4. In addition, activation of vitamin D to 1,25-dihydroxy cholecalciferol also occurs in the placenta, which is an essential element of transplacental phosphate transfer (26). Thus, there is a hypercalcemic status in fetal life due to increased estrogen levels, resulting in enhanced bone modelling and endocortical bone formation (27). All these processes are interupted in preterm neonates, predisposing them to under mineralization of the bone. In addition chronic placental inflammation (chorioamnionitis), or placental insufficiency, as indicated by intrauterine growth retardation, impairs transplacental transfer of calcium and phosphorous creating an osteopenic *milieu* in the fetus. As placental calcium levels and fetal bone accretion depend on maternal dietary calcium intake, calcium supplementation of 2 grams on or after 22 weeks of gestation to pregnant women enhances neonatal BMC (15,26).

### Neonatal Bone Homeostasis

It has been noted that from birth to six months of age, bone physical density is reduced by one third in term neonates (15,27). This results is because of the preferential rapid widening of the bone marrow cavity compared to the cortical surface area. However, term neonates generally maintain bone integrity, unlike preterm infants. There is a fall in transplacentally transferred estrogens and serum calcium levels after birth leading to a rise in PTH (28,29). However, within the first 48 hours of life, falling serum calcium levels do not result in a corresponding rise in serum PTH levels which in turn leads to a nadir in serum calcium levels. It is notable that serum PTH concentrations in term neonates remain within the optimum range for term neonates or adults; they show a falling trend from foetal levels based on measurement in large cohorts of foetuses and neonates not necessarily measured in the same neonates at different points of time (5,15,17).

Calcium absorption in post natal life is a function of type and amount of calcium intake, gastrointestinal function, including both active and passive transport of calcium, and vitamin D levels in the mother. Preterm neonates with reduced intake and inefficient absorption of calcium and phosphorous from the gut are at a twofold disadvantage and are prone to MBD. Oral calcium bioavailability is compromised in cases of large gastric aspirates, vomiting, abdominal distension and constipation, which are often seen in preterm neonates. The interplay of calcium and phosphorous absorption is such that when the dietary levels are disproportionate, one reduces the other’s absorption. Apart from nutritional supplementation, another important factor regulating osteoblastic activity is physical activity during fetal life such as quickening against the uterine wall, which may be lost in sick, preterm neonates who are less active in post natal life. Reduced physical activity enhances osteoclastic activity and inhibits osteoblastic activity, leading to bone resorption and urinary calcium wasting (30,31,32,33).

Interestingly, the *in utero* rise in bone mineral apparent density (BMAD) is faster than that found in *ex utero* babies. BMAD is measured by dividing BMC with the surface area of bone (BMC/BA=g/cm^3^) and is a measure of volumetric BMD. It initially falls after birth but is maintained later on (33). Preterm neonates will have a fall in mineral accretion when compared to fetal life, although skeletal growth remains comparable, and thus leads to osteopenia of prematurity. However, after adequate nutritional supplementation, catch up bone growth begins in preterm VLBW infants.

## Clinical Features and Signs

The clinical manifestations are diverse depending up on the degree of demineralization. MBD can either remain unnoticeable or can present with florid rickets. It can also present as arrested growth velocity and with features of hypocalcemia such as jitteriness or tetany. Affected neonates may have a large head, craniotabes, frontal bossing, sutural separation in the skull, wide fontanelle, costochondral thickening, hypotonia, and protruding abdomen, although this is not consistently present. MBD may manifest with multiple pathological or spontaneous fractures of the ribs and long bones, which is seen in 10% of premature neonates and these present as pain while handling (see Table 2) (3). Rib softening and/or fractures may lead to deranged pulmonary function and respiratory distress around 5 to 11 weeks of age (34,35). These infants can have prolonged ventilator requirement or difficulty in weaning from the ventilator.

## Diagnosis

### Biochemistry

The mainstay of diagnosis is by estimation of biochemical markers which should include serum calcium, phosphorous, PTH and alkaline phosphatase (ALP) and urinary calcium concentrations (see Table 3). The predominant biochemical change includes decreased serum phosphorus levels. Hypophosphatemia is an early indicator of disrupted calcium metabolism and manifests by 7-14 days of life. This can occur either due to isolated phosphate deficiency or to elevated PTH levels. Phosphate depletion increases calcitriol synthesis and may lead to hypercalcemia which supresses PTH levels. In addition phosphate reabsorption is increased by the kidney and thus tubular reabsorption of phosphate is also a useful measure of phosphate homeostasis.

Serum ALP levels ≥900 IU/L show 100% sensitivity and 70% specificity for MBD and ALP concentrations may increase fivefold in MBD (35). Caution should be exercised in interpretation of elevated ALP levels as these may be a symptom of hepatic and/or gastrointestinal diseases because this enzyme is also produced by the liver and gastrointestinal tract. Hence, estimation of the bone iso-enzymeof ALP is more specific for a skeletal cause and thus to diagnose MBD (36,37,38). PTH levels have better specificity than ALP in diagnosing MBD. PTH levels >180 pg/mL or phosphate concentration <4.6 mg/dL at three weeks after delivery have 100% sensitivity and 94% specificity for the diagnosis of severe MBD (6,15,17,36,37,38). Ryan et al (37) in a cohort of 108 preterm neonates, failed to find any association between serum ALP levels and BMC when they reached term.

**Figure 1:**
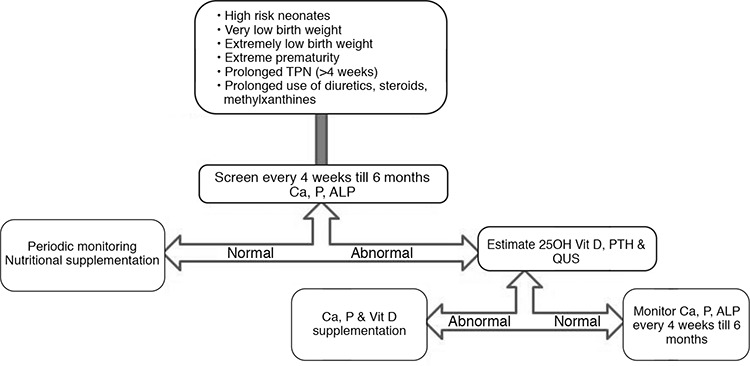
Algorithm for screening of metabolic bone disease ALP: alkaline phosphatase, PTH: parathyroid hormone, TPN: total parenteral nutrition

These biomarkers should be estimated at initial diagnosis and later, during follow-up at four-weekly intervals, to monitor the response to treatment (Figure 1). The fundamental principle in treating these neonates is to establish normocalcemia, normophosphatemia and to prevent urinary calcium wasting. With the normalization of calcium, phosphorus and ALP, evaluation of these parameters should be performed every month up to six months of age and can be done once every three months, thereafter.

### Imaging

Various imaging modalities have been used to diagnose MBD (see Table 3). Plain X-rays will show osteopenia, reduced cortical thickness, rib fractures, widening of the epiphysis, and uneven margins (39). Dual energy X-ray absorptiometry (DEXA) is an imaging tool to detect even small changes in BMC and BMD and to predict the probability of impending fractures. DEXA has been standardized in both term and preterm neonates. Although DEXA has diagnostic precision for bone mineralization, it involves exposure to ionizing radiation and cannot be performed at the bedside (40,41).

Another newer, non-invasive diagnostic modality for MBD is measuring bone speed of sound (SOS) by quantitative ultrasound. This method does not expose to radiation, can be done bedside and has reference standards for both term and preterm infants, both at birth and during follow up. SOS by quantitative ultrasound measures bone density, delineates the structure and enables prediction of bone turnover in preterm infants. This is usually performed using the mid-tibial shaft. Bone SOS is increased in term infants (median 3079 m/s) compared with preterm infants (median 2911 m/s). Similarly, there is a good correlation between gestational age and bone SOS. Also, bone SOS was noted to be low in preterm infants even at a corrected age of 40 weeks when compared with term infants (42,43,44,45).

### Management

**Figure 2:**
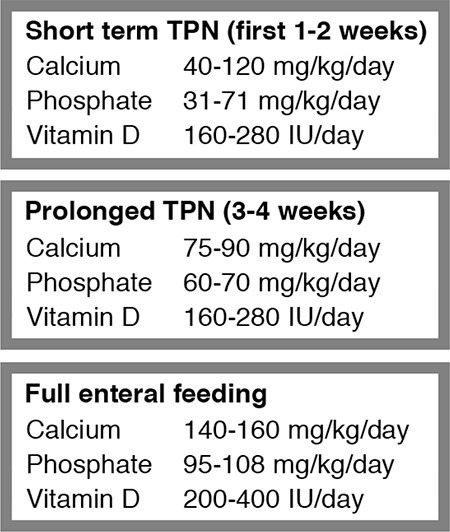
Mineral requrements during total parenteral nutrition and enteral feeds The American Academy of Pediatrics Ref. 29,65,66 TPN: total parenteral nutrition

The principles of management of MBD in preterm neonates are multidimensional (see Figure 2, Table 4) (29).

### Mineral Requirements of Infants

The requirements of calcium and phosphorus are based on intrauterine bone mineral accretion rates. The ideal calcium to phosphorus ratio for optimum skeletal mineralization is 1.7:1 (46). While on PN, use of soluble forms of calcium and phosphorus such as sodium and potassium phosphate, glycerol phosphate or sodium-glucose phosphate, will improve bioavailability (15,21).

Vitamin D requirement is a function of gestational age and maternal vitamin D levels. The fetus is capable of metabolizing vitamin D to 1,25-dihydroxyvitamin D from the 24^th^ week of gestation. It is recommended to provide 400 IU of vitamin D daily for all premature neonates after establishment of full feeds (21).

Calcium and phosphorus requirements in preterm neonates are 123 to 185 mg Ca/100 kcal and 80 to 110 mg P/100 kcal, respectively. This can be achieved with fortification of human milk and with formula milk. Calcium is in the form of soluble calcium glycerol phosphate in formula milk achieving 90 mg/kg/day of calcium absorption (88% of the total). Thus, fortification and supplementation is often mandatory in preterm neonates.

The effects of human milk fortifiers on skeletal mineralization are inconclusive as reported in the Cochrane systematic review and meta analysis of Kuschel and Harding (46). Use of milk fortifiers may predispose to necrotizing enterocolitis with higher doses of calcium, due to increased gastrointestinal transit time, fecal calcium and reduced absorption of fat.

### Prognosis and Outcome

As MBD resolves spontaneously with adequate calcium, phosphorous and vitamin D supplementation, it carries a good prognosis. Although there are differences of opinions about duration, amount and route of mineral supplementation, it has been reported that infants receiving formula feeds until nine months of age have higher BMC (6,15). Also, it has been stated that preterm infants have an adequate catch up by one year of life with optimum supplementation as demonstrated by quantitative ultrasound and DEXA measurements (21,33,39). Skeletal mineralization of term and preterm infants is comparable in later childhood. Similarly, studies have shown reduced spinal BMC in later childhood in LBW neonates who are stunted (3,5,6,15).

Assisted physical exercise is a newer preventive modality which adds to nutritional management in stable premature neonates. Chen et al (43) found that early assisted exercise in VLBW neonates enhanced bone strength. The assisted physical exercise gives either tactile stimulation with moderate pressure strokes or kinaesthetic stimulation with passive flexion and extension of both upper limbs and lower limbs. It was shown to enhance body weight, bone mineralization and osteogenesis. Some studies have shown that exercise may attenuate postnatal reduction in bone SOS (47,48,49,50,51).

It is interesting that nutrition plays a dual role in MBD, both therapeutic and preventive (52,53,54,55,56,57,58). Supplementing mothers with 600 IU/day of vitamin D univerasally has also been shown to help in preventing MBD (59). Focusing on the optimum supply of minerals and of vitamin D, by using human milk fortifier, calcium and phosphorous supplementation or preterm formula is vital to prevent MBD (60,61,62,63,64,65,66).

## Conclusion

Optimum nutritional supplementation of neonates with calcium, phosphorus, and vitamin D, along with assisted physical exercise has been shown to be effective in preventing much MBD. These measures inhibit pathological bone resorption in the initial few weeks of life and enhance the growth of premature infants. It is also vital to identify the biochemical abnormalities characteristic of MBD in a timely manner to initiate therapeutic interventions at the earliest opportunity and thus prevent spontaneous/pathological fractures. Periodic estimation of phosphate and alkaline phosphatase concentrations is important to estimate the risk of osteopenia, along with assessment of treatment efficacy. Similarly, DEXA and quantitative ultrasound enable quantification of bone mineralization and assist in nutritional rehabilitation. Additionally, maternal vitamin D supplementation is another essential preventive strategy for MBD.

## Figures and Tables

**Table 1:**
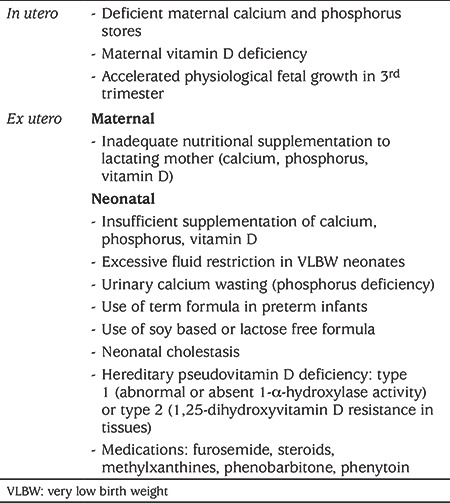
Etiological factors of metabolic bone disease

**Table 2:**
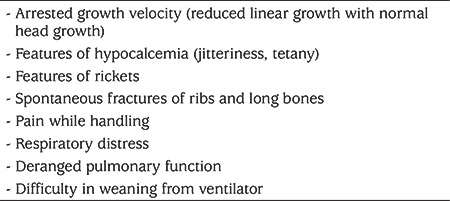
Clinical features of metabolic bone disease

**Table 3:**
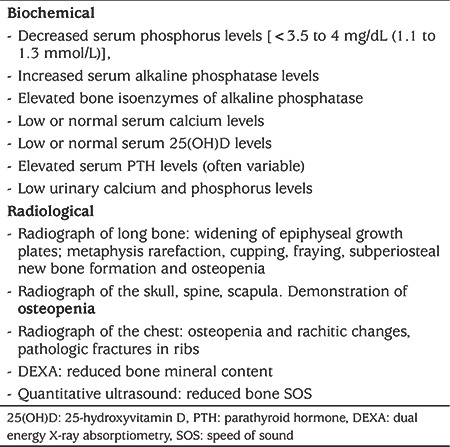
Diagnosis of metabolic bone disease

**Table 4:**
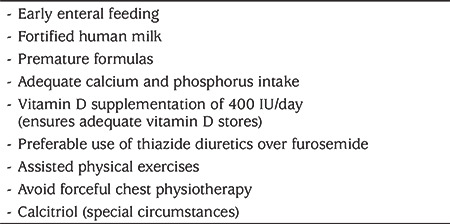
Treatment of metabolic bone disease
